# Recombinants from the crosses between amphidiploid and cultivated peanut (*Arachis hypogaea*) for pest-resistance breeding programs

**DOI:** 10.1371/journal.pone.0175940

**Published:** 2017-04-19

**Authors:** Ailton Ferreira de Paula, Naiana Barbosa Dinato, Bianca Baccili Zanotto Vigna, Alessandra Pereira Fávero

**Affiliations:** 1 Departamendo de Morfologia e Patologia, Universidade Federal de São Carlos, São Carlos, São Paulo, Brasil; 2 Embrapa Pecuária Sudeste, São Carlos, São Paulo, Brasil; Bhabha Atomic Research Centre, INDIA

## Abstract

Peanut is a major oilseed crop worldwide. In the Brazilian peanut production, silvering thrips and red necked peanut worm are the most threatening pests. Resistant varieties are considered an alternative to pest control. Many wild diploid *Arachis* species have shown resistance to these pests, and these can be used in peanut breeding by obtaining hybrid of A and B genomes and subsequent polyploidization with colchicine, resulting in an AABB amphidiploid. This amphidiploid can be crossed with cultivated peanut (AABB) to provide genes of interest to the cultivar. In this study, the sterile diploid hybrids from *A*. *magna* V 13751 and *A*. *kempff-mercadoi* V 13250 were treated with colchicine for polyploidization, and the amphidiploids were crossed with *A*. *hypogaea* cv. IAC OL 4 to initiate the introgression of the wild genes into the cultivated peanut. The confirmation of the hybridity of the progenies was obtained by: (1) reproductive characterization through viability of pollen, (2) molecular characterization using microsatellite markers and (3) morphological characterization using 61 morphological traits with principal component analysis. The diploid hybrid individual was polyploidized, generating the amphidiploid An 13 (*A*. *magna* V 13751 x *A*. *kempff-mercadoi* V 13250)^4x^. Four F_1_ hybrid plants were obtained from IAC OL 4 × An 13, and 51 F_2_ seeds were obtained from these F_1_ plants. Using reproductive, molecular and morphological characterizations, it was possible to distinguish hybrid plants from selfed plants. In the cross between *A*. *hypogaea* and the amphidiploid, as the two parents are polyploid, the hybrid progeny and selves had the viability of the pollen grains as high as the parents. This fact turns the use of reproductive characteristics impossible for discriminating, in this case, the hybrid individuals from selfing. The hybrids between *A*. *hypogaea* and An 13 will be used in breeding programs seeking pest resistance, being subjected to successive backcrosses until recovering all traits of interest of *A*. *hypogaea*, keeping the pest resistance.

## 1. Introduction

Peanut (*Arachis hypogaea* L.) is considered as the fifth largest oilseed crop in the world, after soybean, rapeseed, cotton and sunflower. World production during 2014–2015 was 39.83 million tons [1]. The five major countries producing peanut 2014–2015 were China, India, Nigeria, United States, and Burma. Brazil ranked 17^th^ in production, where approximately 90% of peanut production comes from the state of São Paulo [2, 3].

Pests and foliar diseases are among the factors that mostly limit the economically sustainable production of peanuts in Brazil. The silvering thrips (*Enneothrips flavens* Moulton) and red necked peanut worm (*Stegasta bosquella* Chambers) are considered key pests [4, 5].

Thrips are tiny sucking insects of the order Thysanoptera and usually have between 0.5 and 5.0 mm in length, which when feed, destroy plant cells. The red necked peanut worm is an insect of the order Lepidoptera and usually has between 6.0 and 7.0 mm in length, which feeds on the peanut leaflet while still closed [5, 6].

Peanut crop must be chemically protected from pests to achieve satisfactory yields. Infestations of these insects have an important and peculiar aspect: silvering thrips and red necked peanut worm lodge in the buds (tips) of the branches, causing damage more or less severe to the vegetative growth of plants. This mode of attack requires the use of systemic insecticides for the control of these insects, which are more efficient, but more expensive [5, 6]. The use of resistant varieties is one of the best alternatives for pest control, because they do not harm the environment, keep pests at low levels and reduce costs with pesticides and crop treatment [7].

In the case of peanuts, it is known that many wild species of *Arachis* have resistance to pests, which can be introgressed into cultivars [8]. Obtaining cultivars resistant to pests through plant breeding is an alternative to reduce the cost of production for this crop. Efficient use of exotic peanut germplasm favors research programs aimed at the production of new improved cultivars from germplasm adapted to potential types of resistance to diseases and pests [9].

The main difficulty in the use of wild species in peanut breeding is that the majority of the species of Section *Arachis* are diploids and have genomes A, B, D, F, G or K, while the cultivated species *A*. *hypogaea* is allotetraploid and has genomic formula AABB [10, 11, 12, 13, 14, 15, 16, 17].

To overcome the ploidy barrier between the wild and cultivated peanuts, Simpson [18] and Simpson and Starr [19] showed three forms of gene introgression in *A*. *hypogaea*. The first is the cross between the wild diploid species (2n = 20) with *A*. *hypogaea* AABB (4n = 40), generating a triploid hybrid (3n = 30) which would be treated with colchicine for doubling of chromosomes, making it hexaploid (6n = 60) and fertile. This hexaploid would be backcrossed with *A*. *hypogaea* several times until there is loss of chromosomes, and the progeny again has 40 chromosomes. The second introgression process would be the doubling of chromosomes from wild species with genome A and B (2n = 20) making them tetraploid AAAA and BBBB (4n = 40), with subsequent cross between them, producing a hybrid AABB (4n = 40), which would be crossed with *A*. *hypogaea* AABB (4n = 40). The third method would be the cross of a species with genome A (2n = 20) with a species with genome B (2n = 20), generating a sterile hybrid AB (2n = 20), which would be tetraploidized with colchicine, becoming a fertile amphidiploid AABB (4n = 40) that would be crossed with *A*. *hypogaea* AABB (4n = 40) and backcrossed several times until all the traits of interest in *A*. *hypogaea* are recovered. The third way showed the most promising results by producing an amphidiploid (AABB) and crossing it with cultivated peanut *A*. *hypogaea* AABB (4 n = 40), as was done in this study. The peanut cultivar COAN showing high resistance to root-knot nematodes (*Meloidogyne arenaria* and *M*. *javanica*), was obtained from crosses between *A*. *batizocoi* x *(A*. *cardenasii* x *A*. *diogoi)* by Simpson and Starr [19]. The hybrid of this cross was sterile and was treated with colchicine for chromosome doubling. This amphidiploid was crossed with *A*. *hypogaea* cv. Florunner, generating a hybrid registered as TxAG-6. After five backcrosses and successive selection for agronomic traits and resistance to nematodes, it was released as the cultivar COAN.

Accessions of present study, *A*. *magna* V 13751 and *A*. *kempff-mercadoi* V 13250 showed tolerance to thrips and rednecked peanutworm [8]. Crosses between the accessions of *A*. *magna* V 13751 (female parent, genome B) and *A*. *kempff-mercadoi* V 13250 (male parent, genome A) were performed and individuals of progenies were analyzed by reproductive and morphological characterizations. Two diploid hybrid plants were obtained (Paula et al., unpublished data).

In this context, this study aimed to produce a new amphidiploid from wild species of *Arachis* with distinct genomes, cross the new amphidiploid with an elite cultivar of *A*. *hypogaea*, and perform morphological, molecular and reproductive characterization of the progenies.

## Material and methods

### Development of amphidiploid

The crosses between *A*. *magna* V 13751 (female parent) and *A*. *kempff-mercadoi* V 13250 (male parent) were performed manually in a greenhouse at Embrapa Southeast Livestock (São Carlos, state of São Paulo, Brazil) from December to March, 2010/2011. Flower buds of the female parents were emasculated in the late afternoon and flowers pollinated in the morning the next day. To calculate the percentage of success (PS) of hybridization, the following formula was used: PS = (number of hybrids/number of pollinations) x 100.

During the 2011–2012 growing season, fifteen cuttings of approximately 20 cm were taken from the hybrid *A*. *magna* V 13751 x *A*. *kempff-mercadoi* V 13250 (Ferreira et al., unpublished data), with the aid of scissors. Only the apical leaves still closed were kept and the apexes of cuttings were immersed into test tubes containing 0.2% colchicine. The tubes were closed and placed in BOD incubator (Biochemical Oxygen Demand), using fluorescent white light and 28°C for eight hours. After eight hours, the cuttings were washed in running water for about 20 minutes [20, 21, 22, 23]. Thus, with the aid of a scalpel, the cuttings were cut in a bevel (obliquely) over the last node and planted in plastic cups (180 mL) with the same substrate of pots to develop. At this stage, the cuttings were covered with plastic bags to minimize water loss. When cuttings rooted and grew, the plants were transplanted in pots.

During the growing season of 2012–2013, concurrently to the second crossing season, we analyzed the polyploidization of *A*. *magna* V 13751 x *A*. *kempff-mercadoi* V 13250. AB genome diploid plants usually do not produce seeds, sometimes nor flowers. So, they were considered sterile. Those sterile diploid hybrids with the colchicine treatment, showing presence of “peg” and the appearance of seeds were confirmed as successful amphidiploid. The progeny was evaluated by means of reproductive and morphological characterizations.

### Crosses between *A*. *hypogaea* and amphidiploid

Cultivar IAC OL4 (*A*. *hypogaea* subsp. *hypogaea*) as female parent was crossed with the amphidiploid An 13 (*A*. *magna* V 13751 x *A*. *kempff-mercadoi* V 13250)^4x^ as male parent during 2012/2013 growing season. Reproductive, morphological and molecular characterization was carried out on resultant progenies during 2013–2014 growing season.

### Seed germination

All seeds were treated with Thiram + Distilled water (1:2) for two minutes and placed on germitest paper soaked in 0.65% Ethrel for germination. Germination conditions were 16 h at 20°C in the dark and 08 h at 35°C under fluorescent light. Disposable glasses of 180 mL were prepared with substrate and perforated bottom to receive the newly germinated seeds. Plants remained in the cups to achieve size and vigor to be transplanted to pots with volume of 25x40x40 cm.

### Reproductive characterization

**Fig 1:**
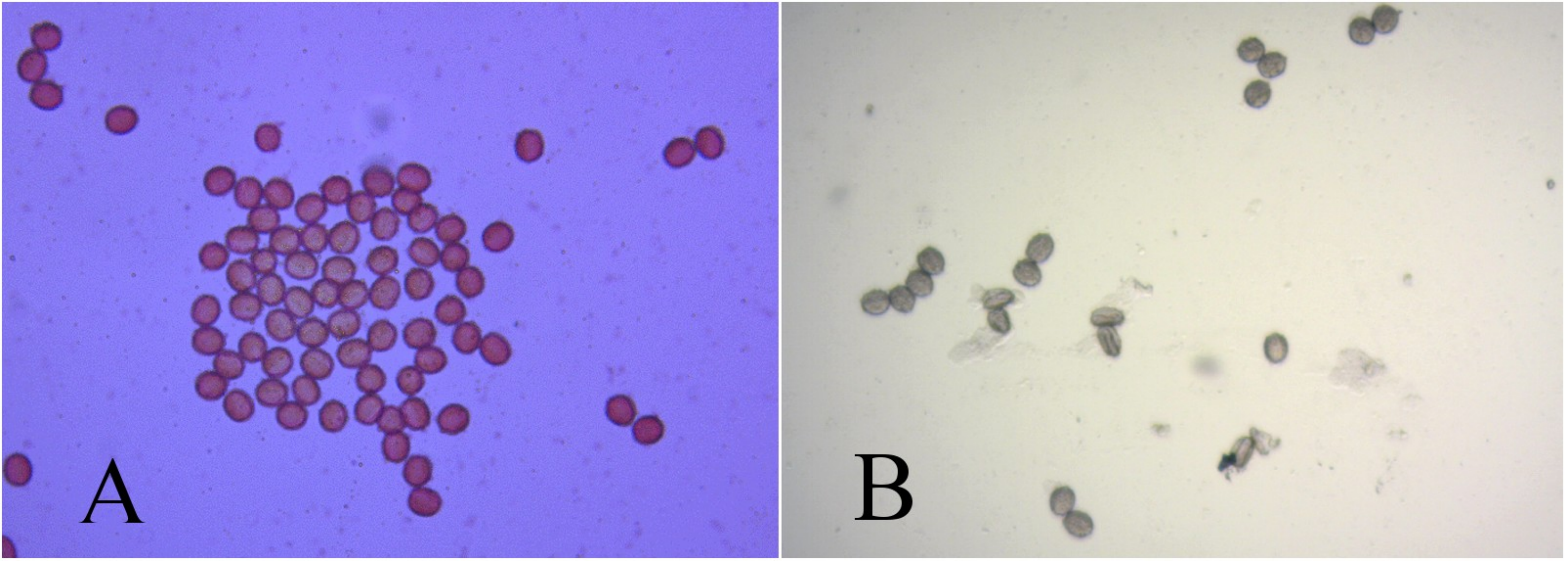
Pollen grains of the hybrid IAC OL4 x An 13. A) Pollen grains stained with 2% carmine acetic acid with glycerin. B) Pollen grains stained with 0.25% tetrazolium. Arrows indicate inviable grains. 100 X magnification under an optical microscope.

Four flowers of each individual used in the research were randomly collected. With the aid of tweezers, pollen grains were taken from the anthers, placed on slides and stained with 2% carmine acetic acid with glycerin (CA) or 0.25% tetrazolium (TZ) for three minutes. Slides were analyzed for viable and non-viable grains under a microscope. Pollen grains were considered as viable when they showed proper development and complete staining (Fig 1). Two hundred grains in each sample (repetition) derived from a single flower, totaling 800 grains per individual were counted. The percentages of stained pollen grains were calculated for each sample of all individuals involved. Analyses of variance and Tukey’s test for means comparison were performed using the software Statistical Analysis System (SAS).

### Morphological characterization

Four leaves of the side branch, a leaf of the main axis and four flowers of all individuals were randomly collected. The leaf collected was always the last leaf expanded of both lateral branches and of the main axis.

Sixty-one morphological traits were analyzed (Table 1), which have been previously evaluated in other studies [21, 22, 23]. According to the trait, the material was measured by ruler or caliper, or observed under a stereomicroscope.

**Table 1:** Descriptors evaluated in genotypes of *Arachis*, respective codes, unit of measurement and in which structure of the plant it was evaluated.

Descriptors	Codes	Unit	MA^2^	LB^3^	Fl^4^
Proximal leaflet length	Pll	Millimeter	X	X	-
Proximal leaflet width	Plw	Millimeter	X	X	-
Distal leaflet length	Dll	Millimeter	X	X	-
Distal leaflet width	Dlw	Millimeter	X	X	-
Petiole length	Pl	Millimeter	X	X	-
Petiolule length	Pol	Millimeter	X	X	-
Lenght of free part of stipule	Lfps	Millimeter	X	X	-
Width of free part of stipule	Wfps	Millimeter	X	X	-
Lenght of adnate part of stipule	Laps	Millimeter	X	X	-
Trichomes on the abaxial leaflet border	Tablb	Scale 1 to 3^1^	X	X	-
Trichomes on the abaxial leaflet center	Tablc	Scale 1 to 3^1^	X	X	-
Trichomes on the abaxial leaflet midvein	Tablm	Scale 1 to 3^1^	X	X	-
Trichomes on the adaxial leaflet border	Tadlb	Scale 1 to 3^1^	X	X	-
Trichomes on the adaxial leaflet center	Tadlc	Scale 1 to 3^1^	X	X	-
Trichomes on the adaxial leaflet midvein	Tadlm	Scale 1 to 3^1^	X	X	-
Bristles on the leaflet border	Blm	Scale 1 to 3^1^	X	X	-
Trichomes on the petiole	Tp	Scale 1 to 3^1^	X	X	-
Trichomes on the petiolule	Tpo	Scale 1 to 3^1^	X	X	-
Bristles on the petiole	Bp	Scale 1 to 3^1^	X	X	-
Bristles on the petiolule	Bpo	Scale 1 to 3^1^	X	X	-
Trichomes on the stipule (free part) center	Tfpsc	Scale 1 to 3^1^	X	X	-
Trichomes on the stipule (free part) border	Tfpsb	Scale 1 to 3^1^	X	X	-
Trichomes on the stipule (adnate part) center	Tadpsc	Scale 1 to 3^1^	X	X	-
Trichomes on the stipule (adnate part) border	Tadpsb	Scale 1 to 3^1^	X	X	-
Bristles on the stipule (free part)	Bfps	Scale 1 to 3^1^	X	X	-
Bristles on the stipule (adnate part)	Badps	Scale 1 to 3^1^	X	X	-
Anthocyanin in the stipule	As	Absence or presence	X	X	-
Standard length	Sl	Millimeter	-	-	X
Standard width	Sw	Millimeter	-	-	X
Wing length	Wl	Millimeter	-	-	X
Wing width	Ww	Millimeter	-	-	X
Lower lip length	Lll	Millimeter	-	-	X
Upper lip length	Ull	Millimeter	-	-	X
Hypanthium length	Hl	Millimeter	-	-	X

^1^(1) Absence, (2) Few, (3) Many

^2^MA: Main axis

^3^LB: lateral branch

^4^FL: flower.

Data were analyzed using Principal Component Analysis (PCA) generated by SAS software. The results of components 1 and 2 were multiplied by the mean values of each trait for each individual and the resulting values were used to construct a Biplot graph using software Microsoft Excel.

### Molecular characterization

Total genomic DNA was isolated from young leaves using the protocol based on CTAB (*Cationic Hexadecyl Trimethyl Ammonium Bromide*) described by Grattapaglia and Sederoff [24], with the inclusion of an additional precipitation with 1.2 M NaCl, immediately after CTAB buffer. Quantification of total DNA was performed with a spectrophotometer (NanoDrop ND-1000).

Three microsatellite markers were pre-selected from a larger set of markers according to the polymorphism in the genitors and to its amplification profile, and were evaluated in this study (Table 2), as follows: Seq3D09, IPAHM406 and RI2A06 [25, 26, 27, 28].

**Table 2:** Microsatellite markers used in this study.

SSR	Amplification temperature °C	Size (pb)	References
IPAHM-406	59	202	Cuc et al., 2008
seq3D9	58	168	Ferguson et al., 2004
RI2A06	52	159	Moretzsohn et al., 2004

Amplification conditions of markers were established from testing under different annealing temperature of primers in the polymerase chain reaction (PCR). PCR reactions were performed in a thermocycler (BioRad T100), with a final volume of 15 μl, as follows: 120ng of genomic DNA, 0.65U Taq DNA polymerase, 1x PCR buffer (200 mM Tris pH 8.4, 500 mM KCl), 1.5 mM MgCl2, 0.2 μM dNTP, and 0.165 μM of each primer. The protocol used for amplification consisted of 95°C for 5 min, 30 cycles (94°C for 45 sec; X°C for 45 sec.; 72°C for 45 sec.), and 72°C for 10 min, where X°C is the specific annealing temperature of the primers. Fragments were visualyzed on 2.5% agarose gel and markers that succeeded in amplification were subjected to electrophoresis in 6% polyacrylamide gels and stained with silver nitrate [29] for visualization of the fragments and genotyping using the 10 bp ladder (Invitrogen). Hybrids were considered those individuals F_1_ who had an allele from the male parent which was not present in the female parent in the evaluated polymorphic loci. As peanut is able to perform self-fertilization, the individuals F_1_ who had only alleles from the female parent and did not have alleles from the male parent were considered as self-plants.

## Results and discussion

### Development of amphidiploid

A total of 105 pollinations were performed between the *A*. *magna* V 13751 x *A*. *kempff-mercadoi* V 13250, which produced seven pegs and two seeds. The two seeds were germinated and were transplanted into pots. Ten cuttings were collected from each hybrid of *A*. *magna* V 13751 x *A*. *kempff-mercadoi* V 13250 (15), totaling 20 stakes treated with colchicine.

The 20 cuttings of the hybrid *A*. *magna* V 13751 x *A*. *kempff-mercadoi* V 13250 (15) treated with colchicine have developed up and just one seed was obtained that produced a plant and was called as An 13 (*A*. *magna* V 13751 x *A*. *kempff-mercadoi* V 13250)^4x^. The presence of seed indicates successful production of amphidiploid. This amphidiploid, An 13 produced a few amount of seeds. For this reason, this amphidiploid was not evaluated in field for pest resistance, as it was done for its progenitors (*A*. *magna* V 13751 and *A*. *kempff-mercadoi* V 13250).

### Development of hybrid between the cultivar IAC OL4 and an amphidiploid An 13

Hybridization between cultivar IAC OL4 and an amphidiploid An 13 was undertaken with 98 pollinations which resulted in 10 pegs in turn 10 seeds. All these 10 seeds were germinated and were planted in cups and transplanted into pots. Among the 10 plants, four were characterized as hybrids and they produced 51 F_2_ seeds with percentage of success of 4.08%. The techniques of emasculation and pollination are among the factors that can influence the percentage of success [30]. Besides these techniques, according to Tallury et al. [31], fertilization itself is difficult and may not occur after pollination or occur late (delayed development of the pollen tube). Problems, such as inability of proembryo to grow after the peg reaches the ground or very slow growth of proembryo may be present.

In a peanut breeding program, for the production of amphidiploid, it is necessary to perform crosses between wild species of *Arachis* which results in sterile interspecific hybrids AB (2n = 20). It is common in these crosses the presence of abortions of “peg” that did not develop seeds, developed seeds that did not germinate and germinated seeds that did not had the vigor to survive in pots [21, 22, 23]. Further, greater number of abortions was due to interspecific hybrids with difference between genomes. In this study, the pollination difficulties cited by Nigam et al. [30] and Tallury et al. [31] are still present, but the problem of the difference between genomes was solved to obtain the amphidiploid. Thus, when performing crosses between *A*. *hypogaea* and amphidiploid with AABB genome, the number of abortions recorded was smaller in comparison with crosses between wild species. This also influences the observed percentage of success. Fávero [21] selected 14 combinations with species with genome A and B in order to obtain interspecific hybrids AB and 2,234 pollinations were performed, which generated 21 plants confirmed as hybrid, reaching a percentage of success of 0.9%. In the same study, when crosses between *A*. *hypogaea* and amphidiploid were conducted, there were 1,359 pollinations, which resulted in 107 hybrid plants, and the success percentage was 7.8%.

### Reproductive characterization

ANOVA evidenced significant differences between individuals, but there were no differences between repetitions in the two tested stainings (CA and TZ) (Table 3, S1 Table).

**Table 3:** Viability of pollen grains of 22 individuals analyzed by staining

Identification	Individual	Mean percentage viability of pollen grains according to the staining^1^	
		CA^2^	TZ^3^
14	V 13250	98.00 a	89.00 ab
6	OL4 x AN13	97.50 a	88.00 ab
10	OL4 x AN13	96.75 a	94.00 a
13	V 13751	96.75 a	77.75 b
8	OL4 x AN13	96.25 a	94.75 a
11	IAC OL4	95.75 a	93.75 a
7	OL4 x AN13	95.50 a	95.75 a
9	OL4 x AN13	94.00 a	96.50 a
5	OL4 x AN13	92.50 a	96.00 a
4	OL4 x AN13	91.25 a	87.25 ab
2	OL4 x AN13	84.75 b	80.75 b
3	OL4 x AN13	82.50 bc	89.50 ab
1	OL4 x AN13	80.75 bc	86.50 ab
12	An 13	77.00 c	88.25 ab
15	V 13751 x V 13250	1.25 d	0.75 c
CV %		2.79	4.96

^1^Means followed by same letters are significantly equal at 5% probability by Tukey’s test

^2^ 2% carmine acetic acid with glycerin

^3^0.25% Tetrazolium solution.

The diploid hybrid *A*. *magna* V 13751 x *A*. *kempff-mercadoi* V 13250 (15) and the progenitors were included in the reproductive characterization to compare them with amphidiploids and the F_1_ hybrids with *A*. *hypogaea*. The parents had a high percentage of pollen grains stained, the female parent *A*. *kempff-mercadoi* V 13250 (plant 14) showed 98.00% pollen grains stained with CA and 89.00% with TZ; the male parent *A*. *magna* V 13751 (13) showed 96.75% with CA and 77.75% with TZ. The hybrid *A*. *magna* V 13751 x *A*. *kempff-mercadoi* V 13250 (15), different from parents, showed low values of pollen grains stained, 1.25% with CA and 0.75% with TZ.

On the other hand, the amphidiploid An 13 (12) presented high viability of pollen grains, both with CA (77.00%) and with TZ (88.25%) (Table 3).

During cell division, colchicine acts as an antimitotic agent, binding to tubulin dimers, preventing the formation of microtubules and consequently the formation of spindle fibers [32]. During mitosis, when the achromatic spindle is damaged or absent, no separation of the duplicated chromosomes in anaphase, and consequently, cell division does not occur and the cell starts a new cell cycle with amount of DNA duplicated [33]. Thus, colchicine induces polyploidy, cells start to have homologous chromosomes, and the problem of irregular meiosis in diploid interspecific hybrids is solved, leading the plant to produce viable pollen grains.

With the problem of irregular meiosis solved, when making the reproductive characterization by means of pollen grain staining, the amphidiploid shows high viability of pollen grains, being largely different from the sterile diploid hybrid, which has low viability of pollen grains. Thus, reproductive characterization is able to clearly identify this sterile hybrid diploid and an amphidiploid.

A high viability of pollen grains was observed due to CA (over 76%) and TZ (over 77%) staining in the hybrids obtained between IAC OL4 and an amphidiploid An 13 and parents (Table 3).

The pollen staining technique can be used to identify diploid interspecific hybrids, due to the high number of non-viable pollen grains. When the diploid interspecific hybrid plants are treated with colchicine, they become polyploid (amphidiploid). These amphidiploids have homologous chromosomes of the two distinct genomes (stable genetic material) and their pollen grains become viable and the plant fertile. In crossing a cultivar with an amphidiploid, the two individuals have stable genetic material with homologous chromosomes. Their progeny will also have a stable genetic material and there is no difference between the parents and the offspring with respect to the viability of pollen grains. Therefore, in this case, pollen staining technique as a method for identification of hybrids is not conclusive. Hence, it is necessary to use other methods such as morphological and molecular characterizations.

A cytogenetic study conducted by Stalker [10] reported the viability of pollen grains associated with meiosis in intra- and interspecific crosses. When the genetic material was stable, the viability showed 90% stained pollen grains with low presence of univalent (below 0.05 univalent) and high rate of bivalent (approximately 10 bivalent) during meiosis. When the genetic material was unstable, that is, without homologous chromosomes, there was low viability of pollen grains, high number of univalent and low amount of bivalent.

The diploid interspecific hybrid has two distinct genomes in the cells with occasional or total absence of chromosome pairing. Given the low genetic similarity between chromosomes of different genomes, the pairing at metaphase I will be compromised, thus causing an irregular meiosis and non-viable pollen grains. *Arachis* species with genome A and species with genome B have around 20% genetic similarity [28].

When considered together the work of Lüdke [23] and Moretzsohn et al. [28], it was possible to establish a relationship between genetic similarity and the viability of pollen grains. Lüdke [23] studied four rounds of intra and interspecific crosses between species of the *Arachis* section, always using accessions of genome B as female parent. They found that the hybrids with the highest average percentage viability of pollen grains were exactly those which had parents with the same genome, in this case, the genome B. The intraspecific hybrid with genome B having the greatest viability of pollen grains was *A*. *valida* V 13514 x *A*. *valida* V 15096 [B x B], which showed 98.40% pollen grains stained with genetic similarity of 58% between the parents. All other intraspecific hybrids had less than 39% genetic similarity between the parents and below 40% viability of pollen grains. As for interspecific hybrids, all combinations presented less than 20% genetic similarity between the parents and all hybrids presented below 4.6% viability of pollen grains. In this context, the lower the genetic similarity between the parents, the smaller the percentage of viability of pollen grains in the produced hybrid.

### Morphological characterization

All individuals were morphologically characterized, including the diploid hybrid *A*. *magna* V 13751 x *A*. *kempff-mercadoi* V 13250 (15) and its progenitors in order to compare them with amphidiploids and the F_1_ hybrids with *A*. *hypogaea* (S2 Table). PCA showed that the three first principal components accounted for 98% of the total variation of morphological traits.

According to the principal component 1, among the 15 most important morphological descriptors explaining the observed variation, five were collected in the main axis of the plant, eight in the lateral branch and two in the flower (Table 4).

**Table 4:** Order of descriptors that contributed most to the morphological variation observed in the principal component 1 (Prin 1) of the Principal Component Analysis of all the individuals evaluated in this work

Order	Descriptors	Codes^1^	Prin 1	Individuals^2^								
				1	2	3	4	11	12	13	14	15
1	Petiolule length MA	PolMA	0.487	39,260	17,630	40,090	14,260	15,550	17,420	13,220	12,620	11,470
2	Petiolule length LB	PolLB	0.487	11,870	6,590	7,980	11,378	11,350	10,850	11,048	10,770	8,355
3	Bristles on the petiole LB	BpLB	0.360	6,110	6,585	6,780	*	7,800	7,650	7,995	7,448	14,263
4	Distal leaflet length MA	DllMA	0.305	44,230	35,670	37,090	63,100	41,900	36,650	41,710	33,530	42,510
5	Distal leaflet length LB	DllLB	0.305	24,430	27,840	34,490	47,978	34,955	30,480	34,423	33,323	32,670
6	Bristles on the petiolule LB	BpoLB	0.230	1,000	1,000	1,000	1,000	1,000	1,000	1,000	1,000	1,000
7	Width of free part of stipule MA	WfpsMA	0.184	2,500	3,290	2,330	2,150	2,150	1,840	1,250	2,410	2,460
8	Width of free part of stipule LB	WfpsLB	0.184	2,950	3,150	3,190	2,510	2,865	2,718	3,160	3,373	4,145
9	Distal leaflet width MA	DlwMA	0.148	20,460	14,910	13,530	28,690	17,640	15,850	17,630	14,820	20,060
10	Distal leaflet width LB	DlwLB	0.148	17,440	17,620	19,830	25,248	21,613	18,345	20,498	20,275	23,055
11	Bristles on the leaflet border LB	BlmLB	0.115	1,000	1,000	1,000	1,000	1,750	1,250	1,500	1,250	1,500
12	Lower lip length FL	BlmFL	0.077	8,080	7,760	8,770	*	7,650	7,720	7,265	8,043	9,520
13	Lenght of adnate part of stipule MA	LapsMA	0.070	12,750	10,090	8,500	11,550	15,290	14,840	17,280	15,220	15,830
14	Lenght of adnate part of stipule LB	LapsLB	0.070	7,280	7,675	8,420	8,773	12,570	10,370	11,045	10,655	7,343
15	Upper lip length FL	UllFL	0.067	6,570	6,745	7,280	*	8,525	7,458	7,575	8,423	8,600

^1^ Codes ending with MA refer to the main axis, with LB, to the lateral branch and with FL, to flower.

^2^1–4: F_1_ hybrids from IAC OL4 x An 13; 11: IAC OL4, 12: An 13; 13: V13751; 14: V13250; 15: V13751 x V13250

* Not evaluated

**Fig 2:**
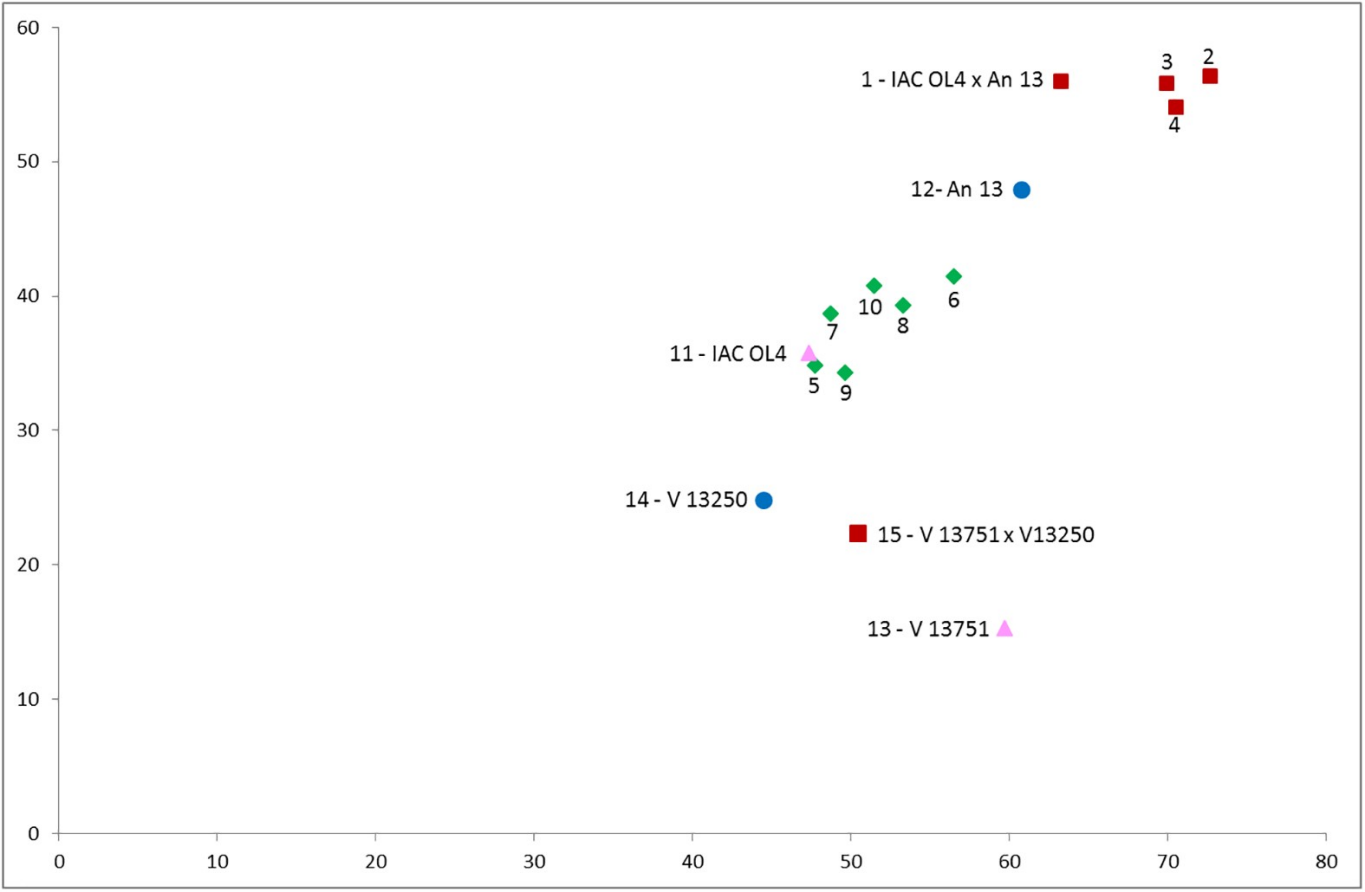
Biplot graph resulting from the cross between IAC OL4 x An 13, obtained by Principal Component Analysis considering the 61 descriptors for the principal components 1 and 2. Triangle indicates female parents V 13751 (13) and IAC OL4 (11); circles, male parents V 13250 (14) and An 13 (12); and representing the progenies, square indicates the hybrid plants V 13751 x V 13250 (15) and IAC OL4 x An 13(1 to 4), and, diamonds, the plants derived from selfing IAC OL4 x An 13 (5 to 10).

From the components 1 and 2, multiplied by the mean values of each trait for each individual, it was possible to construct a biplot graph (Fig 2). The male parent *A*. *kempff-mercadoi* V 13250 (14) was located at the center of the graph and the female parent *A*. *magna* V 13751 (13) was located at the bottom right of the graph, the plant resulting from the cross de *A*. *magna* V 13751 x *A*. *kempff-mercadoi* V 13250 (15) was located closer to the male parent than to the female parent, which allows to characterize this plant as a hybrid by means of PCA.

Analyzing the most important descriptor, it can be observed that the female and male parents, respectively, presented 39.260 and 17.630 mm for the petiolule length of the main axis (PolMA), thus, the hybrid plant *A*. *magna* V 13751 x *A*. *kempff-mercadoi* V 13250 (15) presenting 27.240 mm, was located closest to the male parent. This proximity of the hybrid to the male parent can be also perceived in other descriptors in Table 4.

On the graph, the plant An 13 (amphidiploid) was located far from the diploid plant *A*. *magna* V 13751 x *A*. *kempff-mercadoi* V 13250 (15), indicating morphological variation between these plants (Fig 2). This variation may be explained by the giantism effects observed. Amphidiploid has twice the genetic material in relation to the sterile interspecific hybrid. So, this plant may have an increase in the cell size, which may result in the increasing of size of morphological structures.

With respect to the most important descriptor, it was observed that the hybrid *A*. *magna* V 13751 x *A*. *kempff-mercadoi* V 13250 (15) presented 27.240 mm, and An 13 (12) presented 11.470 mm for the petiolule length of the main axis (PolMA). The difference between the values observed in the descriptors led the hybrid to be located at the bottom right of the graph, and the amphidiploid, at the top right of the graph. Other descriptors and respective measures responsible for the location of these plants on the graph can be seen in Table 4.

It was obtained ten seeds from the cross between IAC OL4 x An 13. Plants of IAC OL4 x An 13 (1, 2, 3 and 4), characterized as a result of hybridization, were located in the top left graph (Fig 2) nearest to the male parent An 13 (12) than to the female parent OL4 IAC (11) on the PCA analysis. The plants IAC OL4 x An 13 (5, 6, 7, 8, 9 and 10) were identified as a result of selfing by molecular paternity test, which were located at the center of the biplot graph, near the female parent IAC OL4 (11). Analyzing the measures of the most important descriptors (Table 4), in most of them, the measurements of hybrids were more similar to the male parent than to the female parent. Thus, the hybrid plants IAC OL4 x An 13 (1, 2, 3 and 4) have measurements that were more similar to the male parent data than to the female parent results. So, they were located closer to the male parent in the graph.

**Fig 3:**
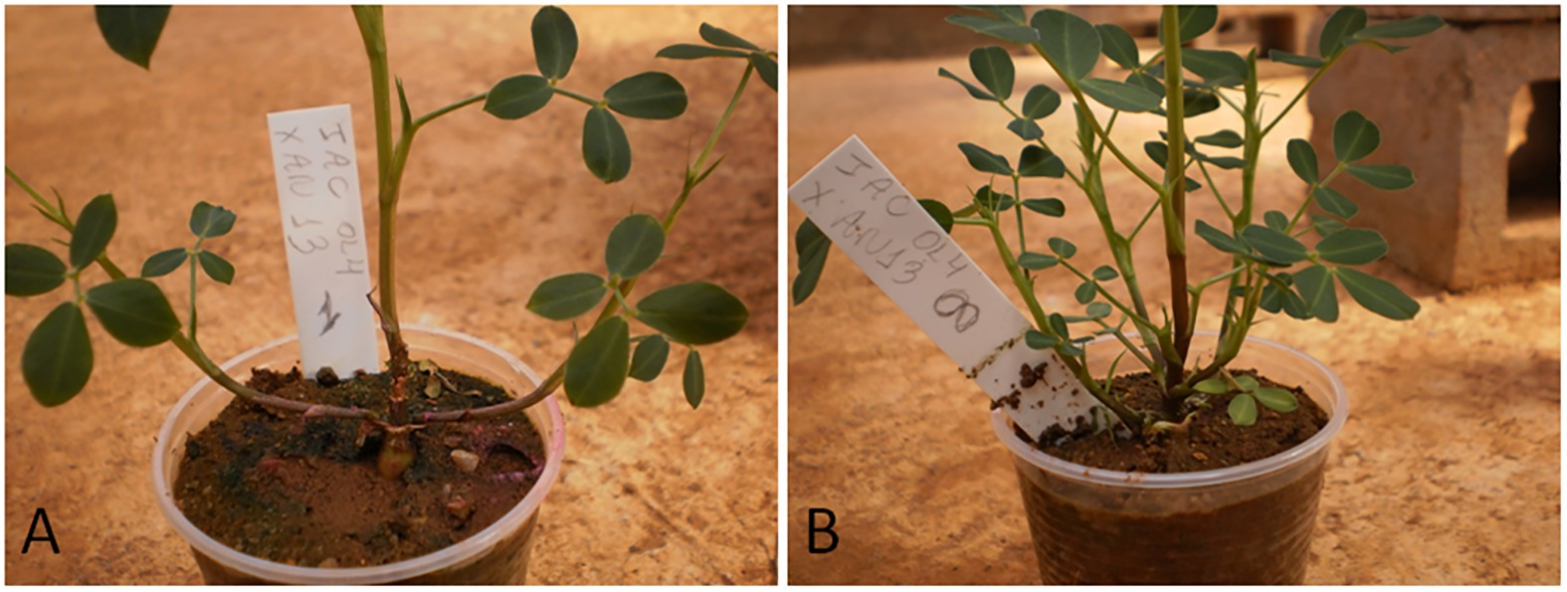
Comparison in the number of lateral branches between plants from. (A) hybridization and (B) selfing.

During the evaluations, the descriptor “number of lateral branches” (nLB), which had no evaluation predicted in the methods, was interesting to identify hybrids between IAC OL4 and amphidiploid An 13. In the growth stage of plants still in disposable cups, four plants (hybrids) presented lower numbers of side branches, compared the other six plants (selfed), which had the same morphological profile of cultivar IAC OL4 (female parent), showing higher number of lateral branches (Fig 3). Thus, at this stage of the breeding program, the descriptor nLB proved efficient to distinguish the hybrid individuals and selfed.

### Molecular characterization of the F_1_ hybrids from *A*. *hypogaea* and An 13

Loci evaluated allowed the identification of hybridization in individuals 1, 2, 3 and 4 and selfing in individuals 5, 6, 7, 8, 9 and 10, all from the cross IAC OL4 x An 13.

**Fig 4:**
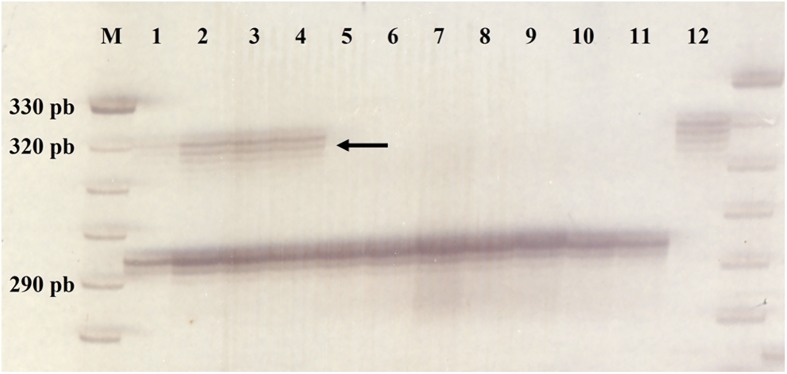
Amplification profile of the marker IPAHM-406 for progenies of IAC OL4 x An 13. Individuals: Hybrids (1 to 4), self-fertilized (5 to 10), female parent IAC OL4 (11) and male parent An 13 (12). Arrow indicates the polymorphic band identifying hybridization. M: 10 bp ladder (Invitrogen).

The Fig 4 shows the amplification profile of the marker IPAHM-406, in which the presence of the 342 bp band in the male parent An 13 (12), and its absence in the female parent IAC OL4 (11) allowed the identification of selfing or hybridization in the progenies. The markers RI2A06 and Seq3d9 showed the same results with respect to the identification of progenies as a result of selfing or hybridization.

Molecular markers are very important tools in molecular characterization of genetic resources and in plant breeding and pre-breeding. In *Arachis*, several studies used molecular markers to characterize germplasm collections, to study genetic diversity [25, 26, 34, 35, 36] and to confirm hybridizations. Fávero [22] using the SSR Lec-1 on 1.2% agarose gel, was able to identify 17 hybrids derived from crosses between KG 30006 x V13710 e KG 30076 x V 12812. Moretzsohn et al. [28] studied the molecular genetic relations between cultivated peanut (*Arachis hypogaea*) and wild species. Thus, it was possible to obtain important information about the genomes and genetic similarity of wild species, which is important to explain the irregular meiosis that diploid interspecific hybrids have, and how this problem is solved by obtaining the amphidiploid. In this study, microsatellites were extremely efficient in identifying hybridization in controlled crosses between IAC OL4 and amphidiploid An 13.

The obtainment of F_2_ plants of an interspecific hybrid that include three very distinct species is the first step for the use of these germplasm in breeding programs. After that it will be necessary the confirmation of the introgression of pest resistance genes in the progenies, select the best ones and backcross these genotypes with *A*. *hypogaea* for at least eight times. Bioassays for pest resistance may be done before each backcrossing season.

#### Observation of mite resistance during evaluations

**Fig 5:**
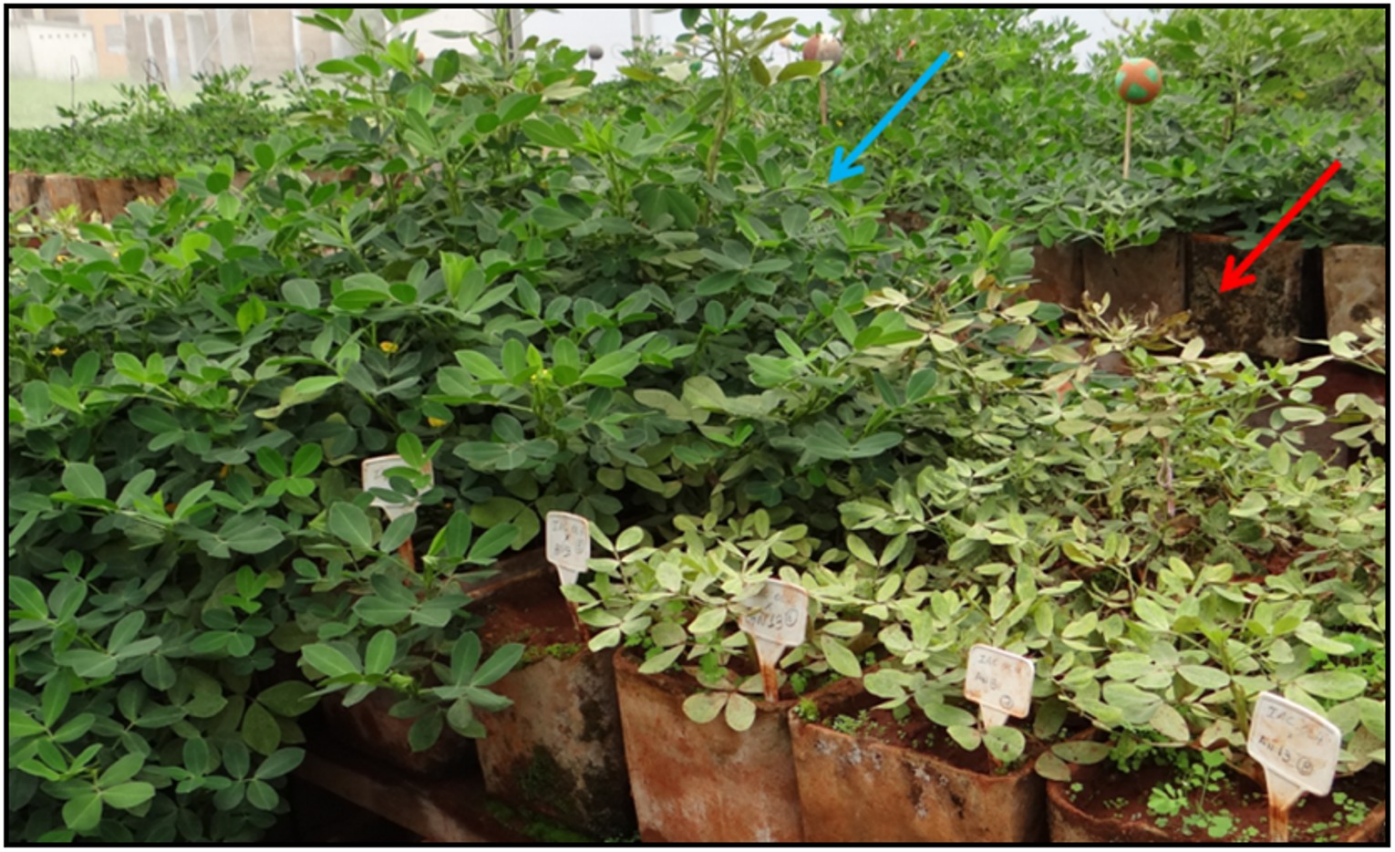
Progeny of IAC OL4 x An 13 under attack by mites. The blue arrow indicates the plants resistant to mite, result from hybridization. The red arrow indicates the plants susceptible to mite, a result from selfing.

**Fig 6:**
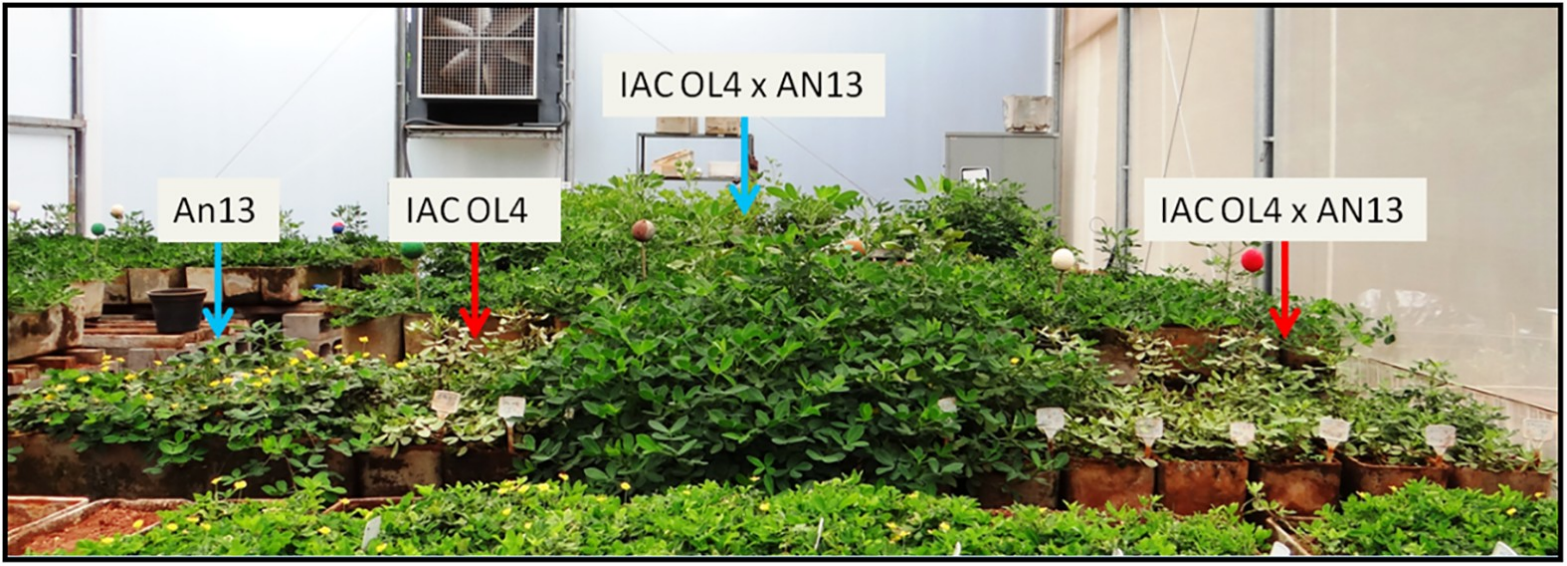
Parents and progeny of IAC OL4 x An 13 under attack by mites. The blue arrow represents the plants resistant to mite (An 13 and hybrid). The red arrow represents the plants susceptible to mite (IAC OL4 and selfed).

At the end of 2014–2015 growing season, parents and progenies plants were attacked by mites (*Mononychellus planki* (McGregor)) in greenhouse. Realizing that the plants An 13 (12) and the hybrids IAC OL4 x An 13 (1, 2, 3 and 4) were resisting the attack, all pots (parents and progenies) were no longer sprayed with acaricide. The female parent IAC OL4 (11) and plants of the progeny of the cross IAC OL4 x An 13 (5, 6, 7, 8, 9 and 10) considered as resulting from selfing suffered such a great pressure from mites that no longer developed and leaves began to yellow (Figs 5 and 6). While the female parent IAC OL4 (11) was attacked by mites, the male parent An 13 (12) and the plants IAC OL4 x An 13 (1, 2, 3 and 4), considered hybrids, continued their development normally (Fig 6). It was observed the presence of mites in hybrid plants, but at a much lower level compared to plants arising from selfing. By observations of plants in this attack, it was possible to identify resistance to mites in hybrids and amphidiploid An 13, and observe that hybrids have resistance that the IAC OL4 does not have.

## 6. Conclusions

In the present study, sterile diploid hybrid was obtained from the cross between the accessions of *A*. *magna* V 13751 (genome B) and *A*. *kempff-mercadoi* V 13250 (genome A) showing resistance to thrips and rednecked peanutworm. The resultant hybrid was polyploidized to obtain an amphidiploid which was crossed with an elite cultivar of *A*. *hypogaea* and F_2_ seeds were generated.

Using the reproductive, molecular and morphological characterizations, it was possible to distinguish hybrid plant individuals from selfing.

In the cross between *A*. *hypogaea* cv. IAC OL4 and amphidiploid An 13, the hybrid progeny had a pollen grain viability as high as that of parents. This prevented the use of reproductive traits for discriminating, in this case, the hybrids of individuals from selfing.

The 51 F_2_ seeds produced by the hybrids IAC OL4 x An 13 will be used in breeding programs, and may confer resistance to thrips and rednecked peanutworm for the cultivated peanut.

## Supporting information

S1 Table Raw data of the reproductive characterization of the 15 *Arachis* genotypes evaluated by Paula et al. * Not evaluated.
(XLSX)

S2 Table Raw data of the morphological characterization of the 15 *Arachis* genotypes evaluated by Paula et al. ^1^See Descriptors details in Table 1. * Not evaluated.
(XLSX)
